# Harnessing microRNA-enriched extracellular vesicles for liquid biopsy

**DOI:** 10.3389/fmolb.2024.1356780

**Published:** 2024-02-21

**Authors:** Song Yi Ko, WonJae Lee, Honami Naora

**Affiliations:** Department of Molecular and Cellular Oncology, University of Texas MD Anderson Cancer Center, Houston, TX, United States

**Keywords:** microRNA, extracellular vesicles, cancer, liquid biopsy, biomarkers, membrane protein, CD147

## Abstract

Extracellular microRNAs (miRNAs) can be detected in body fluids and hold great potential as cancer biomarkers. Extracellular miRNAs are protected from degradation by binding various proteins and through their packaging into extracellular vesicles (EVs). There is evidence that the diagnostic performance of cancer-associated extracellular miRNAs can be improved by assaying EV-miRNA instead of total cell-free miRNA, but several challenges have hampered the advancement of EV-miRNA in liquid biopsy. Because almost all types of cells release EVs, cancer cell-derived EVs might constitute only a minor fraction of EVs in body fluids of cancer patients with low volume disease. Furthermore, a given cell type can release several subpopulations of EVs that vary in their cargo, and there is evidence that the majority of EVs contain low copy numbers of miRNAs. In this mini-review, we discuss the potential of several candidate EV membrane proteins such as CD147 to define cancer cell-derived EVs, and approaches by which subpopulations of miRNA-rich EVs in body fluids might be identified. By integrating these insights, we discuss strategies by which EVs that are both cancer cell-derived and miRNA-rich could be isolated to enhance the diagnostic performance of extracellular miRNAs.

## 1 Introduction

MicroRNAs (miRNAs) are a class of non-coding RNAs of approximately 21 nucleotides in length that regulate gene expression post-transcriptionally (Krol et al., 2010). Expression patterns of miRNAs reflect the developmental lineage and differentiation state of tumors and are highly informative for cancer diagnosis and prognosis (Lu et al., 2005; Volinia et al., 2006; Kim et al., 2011; Dvinge et al., 2013). Extracellular miRNAs are relatively stable and can be detected in body fluids (Mitchell et al., 2008), holding great potential for liquid biopsy. Unlike conventional tissue biopsy, liquid biopsy is minimally invasive and readily repeatable, enabling serial monitoring following surgery and treatment. Extracellular miRNAs are stabilized in body fluids through forming complexes with high-density lipoprotein (Vickers et al., 2011), RNA-binding proteins (RBPs) (Wang et al., 2010; Arroyo et al., 2011), and nano-sized proteinaceous particles (Zhang et al., 2021). Furthermore, cells release miRNAs along with other nucleic acids, proteins, and lipids in enclosed membranous structures called extracellular vesicles (EVs) (Valadi et al., 2007; Maas et al., 2017; Xu et al., 2018).

EVs mediate intercellular communication by acting as delivery vehicles that transfer informational cargo from one cell to another (Maas et al., 2017). There is considerable evidence that EV-mediated transfer of cargo between cancer cells and stromal cells facilitates tumor growth and metastasis (Xu et al., 2018). EVs are ideal for liquid biopsy because EVs contain cargo that often reflects the genetic and biological status of the parental cell, protect their cargo from degradation, and can be detected in body fluids of cancer patients (Xu et al., 2018). Cancer-associated miRNAs have been detected in EVs that were isolated from a variety of body fluids by polymer-based precipitation or by separation based on particle density, size, or surface charge (Rabinowits et al., 2009; Madhavan et al., 2015; Yasui et al., 2017; Wang et al., 2022). However, body fluids contain EVs of diverse cellular origins. Because almost all types of cells release EVs, cancer cell-derived EVs might constitute only a minor fraction of EVs in body fluids of cancer patients with small, early-stage tumors. Furthermore, EVs are elevated in other conditions such as coronary artery disease, hypertension, and diabetes (Bernal-Mizrachi et al., 2003; Preston et al., 2003; Li et al., 2016) that are common comorbidities of cancer patients (Roy et al., 2018). As such, the representation of cancer cell-derived EVs might be low in cancer patients who have comorbid conditions. Methods that enrich for EVs released by cancer cells could therefore enhance the detection of cancer-associated extracellular miRNAs.

**FIGURE 1:**
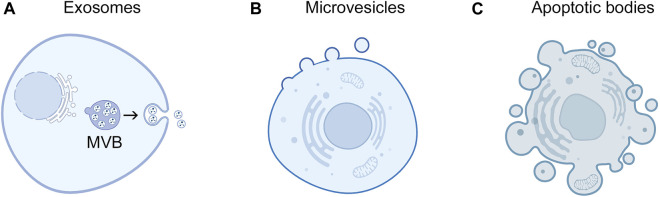
Subcellular origins of EVs. Exosomes and microvesicles are released by live cells. **(A)** Exosomes derive from endosomal compartments called multivesicular bodies (MVB) that contain intraluminal vesicles. Upon fusion of MVBs with the plasma membrane, intraluminal vesicles are released into the extracellular space as exosomes. Exosomes are typically 30 nm–150 nm in diameter. **(B)** Microvesicles form through outward budding and pinching of the plasma membrane and range from 100 nm to 1 µm in diameter. **(C)** Apoptotic bodies are generated though membrane blebbing and fragmentation of apoptotic cells and are typically 1 μm–5 µm in diameter.

Another challenge that can limit the detection of EV-miRNAs is that an individual cell type can release several subpopulations of EVs that vary in their cargo including their miRNA content (Kowal et al., 2016; Temoche-Diaz et al., 2019; Barman et al., 2022). Three broad types of EVs have been described in terms of their subcellular origin: exosomes, microvesicles (ectosomes), and apoptotic bodies (Maas et al., 2017; Xu et al., 2018) (Figure 1). These types of EVs vary in size (Figure 1) but can only be definitively differentiated by real-time high-resolution imaging (Théry et al., 2018). Notably, several studies have identified that EVs contain only a minor fraction of extracellular miRNAs, and that the majority of EVs contain low copy numbers of miRNAs (Wang et al., 2010; Arroyo et al., 2011; Chevillet et al., 2014; Albanese et al., 2021; Zhang et al., 2021). To improve detection of EV-miRNA biomarkers, approaches that can define and isolate subpopulations of intact miRNA-rich EVs in body fluids are needed.

Here, we firstly describe several EV membrane proteins, highlighting their strengths and limitations as surface markers of cancer cell-derived EVs. Secondly, we describe several mechanisms by which miRNAs are encapsulated in EVs and how subpopulations of miRNA-rich EVs might be identified. Finally, by drawing these insights together, we discuss approaches by which EVs that are both cancer cell-derived and miRNA-rich could be isolated to improve the diagnostic efficacy of extracellular miRNAs.

## 2 Distinguishing EVs that derive from cancer cells

EV membrane proteins are ideal for differentiating subpopulations of intact EVs because these proteins reflect their cell-of-origin and can be captured by antibodies. Proteomic analysis has revealed several EV surface markers (Im et al., 2014; Kowal et al., 2016). CD9, CD63 and CD81 are members of the tetraspanin family of membrane proteins and are enriched in MVB and small EVs (Escola et al., 1998; Kowal et al., 2016). EVs have been isolated from body fluids by immunocapture of CD9, CD63 and/or CD81 (Logozzi et al., 2009; Duijvesz et al., 2015; Campos-Silva et al., 2019). However, these tetraspanins are ubiquitously expressed (Maecker et al., 1997). A recent study traced the cellular origin of circulating CD9-positive EVs in mice bearing human cervical and renal cancer xenografts and found that the majority (i.e., approximately 70%) of CD9-positive EVs derive from mouse host cells (Ko et al., 2023).

Epithelial cell adhesion molecule (EpCAM) has been thought to be a candidate marker for enriching cancer cell-derived EVs. EpCAM is expressed at variable levels in normal epithelial tissues but is overexpressed in many types of carcinomas including 94% of colorectal cancers, 46% of invasive ductal breast cancers, 74% of non-small cell lung cancers, 73% of ovarian cancers, 63% of pancreatic cancers, and 89% of prostate cancers (Spizzo et al., 2011). Elevated levels of EpCAM-positive EVs have been detected in body fluids of patients with ovarian, pancreatic, and prostate cancers (Im et al., 2014; Zhao et al., 2016; Amrollahi et al., 2019; Dai et al., 2021). However, the ectodomain of EpCAM can be cleaved from EVs by serum metalloproteinases, thereby hampering the immunocapture of cancer cell-derived EVs from body fluids (Rupp et al., 2011).

CD24 is a mucin-like glycoprotein that is expressed in various types of cancers including 85% of breast cancers, 45% of non-small cell lung cancers, 83% of ovarian cancers, 48% of prostate cancers, and 72% of pancreatic cancers (Kristiansen et al., 2004). However, CD24 is expressed in many types of immune cells and some non-hematopoietic cells (Fang et al., 2010). CD24-positive EVs have been detected in body fluids of patients with breast and ovarian cancers (Rupp et al., 2011; Im et al., 2014; Zhao et al., 2016), but also in healthy subjects (Keller et al., 2007). The cellular origin of CD24-positive EVs in body fluids of cancer patients is unclear and merits investigation.

The proteoglycan glypican-1 has been detected in circulating EVs of pancreatic cancer patients, and glypican-1-positive EVs reportedly distinguish patients with pancreatic cancer from healthy subjects and patients with benign pancreatic disease (Melo et al., 2015). However, glypican-1 is not only strongly expressed in pancreatic cancer cells but also in adjacent fibroblasts (Kleeff et al., 1998; Tsujii et al., 2021). Pancreatic cancer-associated fibroblasts have been found to secrete glypican-1-positive EVs (Nigri et al., 2022). Fibroblasts are a major constituent of desmoplastic stroma that accounts for up to 90% of the volume of pancreatic tumors (Neesse et al., 2011). It is therefore possible that the majority of glypican-1-positive EVs in pancreatic cancer patients might not derive from cancer cells.

A deletion in the epidermal growth factor receptor (EGFR) gene results in a constitutively activated receptor (termed EGFRvIII) and occurs in 25%–64% of glioblastoma multiforme cases (Gan et al., 2013). EGFRvIII has been detected in EVs secreted by EGFRvIII-expressing glioma cells (Lee et al., 2018) and in circulating EVs of patients with high-grade gliomas (Graner et al., 2009). The presence of EGFRvIII in other types of cancer such as non-small cell lung cancer is controversial (Gan et al., 2013). This might explain why a large cohort study of lung cancer patients and non-cancerous subjects failed to validate EGFRvIII as an EV-associated cancer biomarker (Sandfeld-Paulsen et al., 2016).

The glycoprotein CD147 (also known as EMMPRIN or basigin) is one of the most frequently detected proteins in EVs (Ko et al., 2023) and is expressed in at least 25 types of cancers of diverse origin (Supplementary Table S1). CD147 is also expressed in some types of normal cells such as renal tubular epithelial cells, leukocytes, platelets, and endothelial cells (Kosugi et al., 2015). However, it has been shown that renal cancer cells secrete significantly more CD147-positive EVs than normal renal tubular epithelial cells and endothelial cells (Ko et al., 2023). Furthermore, CD147 has been found to be enriched in pancreatic and lung tumor-derived EVs as compared to EVs from normal tissues (Hoshino et al., 2020). Analyses of the cellular origin of circulating EVs in mice bearing human cervical and renal cancer xenografts has revealed that 75%–81% of CD147-positive EVs derive from cancer cells (Ko et al., 2023). Notably, increases in cancer cell-derived CD147-positive EVs were detected in mouse xenograft models from an early stage (Ko et al., 2023). Moreover, significant increases in circulating CD147-positive EVs have been detected in patients with colorectal, ovarian, and renal cancers from the earliest stages of disease (Tian et al., 2018; Ko et al., 2023). CD147 could therefore be a candidate surface marker to identify EV subpopulations that are enriched in cancer cell-derived EVs in multiple disease sites and across all disease stages including early-stage disease.

## 3 Distinguishing EVs that are enriched in miRNA

Early studies revealed that miRNA profiles in EVs vary from those of the parental cell, suggesting that miRNAs are selectively packaged into EVs (Valadi et al., 2007; Skog et al., 2008). Subsequently, several RBPs have been found to control sorting of miRNAs into EVs. Y-box protein 1 has been shown to be required for packaging miRNAs and other small non-coding RNAs into EVs (Shurtleff et al., 2016; Shurtleff et al., 2017). Heterogenous nuclear ribonucleoprotein A2/B1 (hnRNP A2/B1) and synaptotagmin-binding cytoplasmic RNA-interacting protein (SYNCRIP) preferentially sort miRNAs with specific sequence motifs (i.e., GGAG and GGCU, respectively) into EVs (Villarroya-Beltri et al., 2013; Santangelo et al., 2016). The role of Argonaute-2 (Ago2), a component of the RNA-induced silencing complex, in mediating the sorting of miRNAs into EVs has been controversial. Ago2 has been implicated in the sorting of miRNAs into tetraspanin-positive EVs in a manner dependent on KRAS-MEK signaling (McKenzie et al., 2016). However, other studies have found that tetraspanin-positive EVs do not contain Ago2 (Jeppesen et al., 2019), and that extracellular miRNAs associate with non-vesicular Ago2 complexes (Arroyo et al., 2011).

The mechanisms by which RBP-miRNA complexes are incorporated into EVs are not well-understood. There is evidence supporting a role for caveolin-1, a protein enriched in invaginations of the plasma membrane, in guiding RBP-miRNA complexes into EVs. Caveolin-1 has been detected in a subpopulation of miRNA-rich EVs in bronchoalveolar lavage fluid (Lee et al., 2019a). Oxidative stress-induced phosphorylation of caveolin-1 leads to an interaction between hnRNP A2/B1 and caveolin-1 that in turn escorts the hnRNP A2/B1-miRNA complex into EVs (Lee et al., 2019b). It has also been reported that vesicle-associated-membrane-protein-associated protein A (VAP-A), an endoplasmic reticulum (ER)-anchored protein, promotes the biogenesis of RNA-containing EVs at ER membrane contact sites (Barman et al., 2022). Knockdown of VAP-A has been found to significantly reduce the levels of hnRNP A2/B1, SYNCRIP, and Ago2 and the miRNA content in EVs (Barman et al., 2022).

Identifying miRNA-rich EVs within a heterogenous population of EVs has been challenging. A subpopulation of miRNA-rich EVs was identified in bronchoalveolar lavage fluids by isolating total EVs by ultracentrifugation, followed by density gradient fractionation and analysis of RNA content in EVs in each fraction (Lee et al., 2019a). Notably, this miRNA-rich EV subpopulation constituted only 6% of the total EV population (Lee et al., 2019a). By using a similar approach, a miRNA-rich EV subpopulation was isolated from conditioned media of colon cancer cells and constituted only 10% of total EVs (Barman et al., 2022). These findings support the existence of a subpopulation of miRNA-rich EVs and reinforce the rationale for isolating this subpopulation to enhance detection of EV-miRNA biomarkers. However, isolating EVs by density gradient fractionation requires large sample volumes, is labor-intensive, and impracticable for a clinical laboratory setting.

**FIGURE 2:**
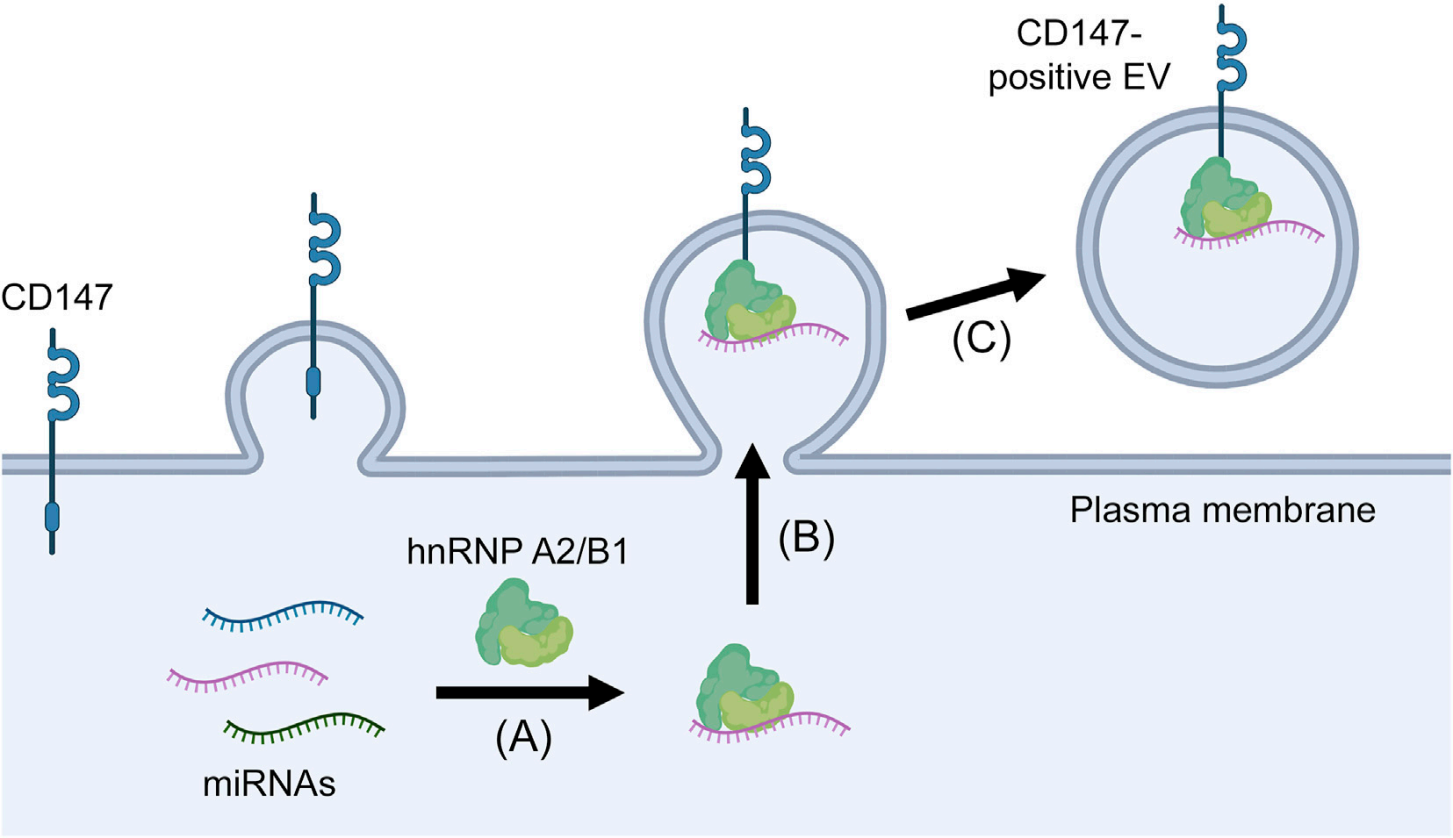
Proposed mechanism of miRNA enrichment in CD147-positive EVs. MiRNAs form complexes with hnRNP A2/B1 **(A)** that in turn are recruited to the plasma membrane through the interaction of hnRNP A2/B1 with CD147 **(B)** and are then released in microvesicles that pinch off from the cell surface **(C)**.

A recent study proposed CD147 as a surface marker that can define miRNA-rich EVs (Ko et al., 2023). This study initially identified CD147-positive EVs as a subpopulation that is distinct from tetraspanin-positive EVs. CD147-positive EVs are likely to be microvesicles because CD147 mostly localizes to the plasma membrane and CD147-positive EVs are generated independently of the Endosomal Sorting Complex Required for Transport machinery that controls exosome biogenesis (Ko et al., 2023). Notably, analysis of EVs in plasma of ovarian and renal cancer patients and of EVs released by cancer cells revealed that CD147-positive EVs have an 8- to 26- fold higher miRNA content than tetraspanin-positive EVs (Ko et al., 2023). HnRNP A2/B1 was not detected in tetraspanin-positive EVs as similarly reported by other investigators (Jeppesen et al., 2019), but was enriched in CD147-positive EVs (Ko et al., 2023). Immunoprecipitation assays revealed that CD147 interacts with hnRNP A2/B1, and the miRNA content in CD147-positive EVs was substantially reduced when hnRNP A2/B1 was knocked out (Ko et al., 2023). These findings implicate that CD147-positive EVs are selectively enriched in miRNA through the interaction of CD147 with hnRNP A2/B1 (Figure 2), and raise the possibility that miRNA-rich EVs in body fluids of cancer patients can be isolated by CD147 immunocapture.

## 4 Discussion

Extracellular miRNAs hold great potential as biomarkers for cancer diagnosis, prognosis, and recurrence. These miRNAs are protected against ribonucleases through their encapsulation in EVs or association with non-vesicular protein complexes, and can be detected in a variety of body fluids. Typically, total cell-free miRNA is isolated from body fluids by using organic solvents or silica-based columns (El-Khoury et al., 2016). However, trace amounts of miRNAs derived from small tumors may evade detection. Several studies have shown that assaying EV-miRNA improves the diagnostic performance of cancer-associated miRNAs. The ability of several miRNAs to differentiate patients with prostate cancer and with benign prostatic hyperplasia was increased by assaying EV-miRNA as compared to total cell-free miRNA (Endzeliņš et al., 2017). Similarly, a case-control study of patients with early-stage colon cancer found that the diagnostic efficacy of cancer-associated miRNAs was higher using EV-miRNA than total cell-free miRNA (Min et al., 2019).

Given that almost all types of cells release EVs and that the majority of EVs contain low copy numbers of miRNAs (Chevillet et al., 2014; Albanese et al., 2021; Zhang et al., 2021), EV isolation methods that enrich for EVs that (a) are released by cancer cells and (b) are also miRNA-rich would be the optimal approach to enhance the diagnostic performance of cancer-associated miRNAs. With the exception of mutated antigens such as EGFRvIII, most proteins that have been proposed as ‘cancer EV markers’ are overexpressed in tumors and at variable levels in normal cells, and the cellular origins of EVs that express these markers require clarification. Of the candidate markers, CD147 has several advantages including its prevalent expression in cancers of diverse origin (Supplementary Table S1), its enrichment in cancer cell-derived EVs (Hoshino et al., 2020), and evidence that circulating CD147-positive EVs predominantly derive from cancer cells and are significantly elevated in patients with colorectal, ovarian and renal cancers from the earliest stages of disease (Tian et al., 2018; Ko et al., 2023). However, validation of these findings in large cohorts and in multiple disease sites is needed.

A substantial advantage of CD147 is that it can also define a subpopulation of EVs with high miRNA content. To the best of our knowledge, no other surface marker of miRNA-rich EVs has been identified. Because CD147-positive EVs predominantly derive from cancer cells, CD147 immunocapture might be an ideal method to increase the sensitivity of detection of cancer-derived extracellular miRNAs. Notably, it has been found that extracellular miRNAs isolated by CD147 immunocapture from body fluids of patients with ovarian and renal cancers more closely reflect the miRNA signatures of matching tumor tissues than total cell-free miRNA (Ko et al., 2023). Furthermore, plasma levels of miR-210, a widely studied biomarker of renal cell carcinoma (Zhao et al., 2013; Dias et al., 2017) could effectively differentiate patients with early-stage renal cell carcinoma and healthy subjects when extracellular miRNAs were isolated by CD147 immunocapture but not when total cell-free miRNA of the same cohort was isolated (Ko et al., 2023). These findings indicate that isolating extracellular miRNAs by CD147 immunocapture can improve the diagnostic performance of miRNA biomarkers.

Cancer cells may release other subpopulations of EVs that are miRNA-rich. It has been found that cancer cells release a distinct subpopulation of CD98-positive EVs that have a miRNA content higher than that of tetraspanin-positive EVs but lower than that of CD147-positive EVs (Ko et al., 2023). In contrast to CD147-positive EVs, hnRNP A2/B1 was not detected in CD98-positive EVs (Ko et al., 2023). Distinct sets of miRNAs might be differentially sorted into these EV subpopulations because hnRNP A2/B1 has been reported to preferentially sort miRNAs with a GGAG motif into EVs (Villarroya-Beltri et al., 2013). Although CD147 interacts with hnRNP A2/B1 (Ko et al., 2023), it is unclear whether this occurs through direct binding. CD147 might interact with hnRNP A2/B1 through caveolin-1 because CD147 associates with caveolin-1 (Tang and Hemler, 2004) and hnRNP A2/B1 mediates sorting of miRNAs into EVs by interacting with caveolin-1 (Lee et al., 2019b).

In summary, as circulating carriers of miRNA and other informational cargo, EVs are ideal for liquid biopsy. However, greater rigor and reproducibility are needed. Variations in methods of processing and storing body fluids, isolating EVs, and extracting, detecting, and normalizing miRNA levels have contributed to discordant findings (Witwer et al., 2013; Buschmann et al., 2018; Coenen-Stass et al., 2018). The minimal information for studies of extracellular vesicles (MISEV) is a field-consensus initiative of the International Society for Extracellular Vesicles that is directed to improving rigor and standardization in EV research (Théry et al., 2018). Adoption of MISEV guidelines and robust standardized methods will enable more reliable validation of EV-miRNAs.
