# Supplementary material for: Harnessing microRNA-enriched extracellular vesicles for liquid biopsy
Source: Front Mol Biosci. 2024 Feb 21;11:1356780. doi: 10.3389/fmolb.2024.1356780 (PMC10916008; doi:10.3389/fmolb.2024.1356780)
Supplement: Supplementary file 1 [file DataSheet1.PDF]

Supplementary Table 1. CD147 expression in human cancers

Cancer type	Subtype	Sample size	CD147-positive cases (%)	Reference
Bladder cancer	Transitional cell carcinoma	53<br>108<br>360	35.8<br>73.1<br>63.3	Riethdorf et al.,2006<br>Xue et al.,2011<br>Choi et al.,2014
Bone cancer	Osteosarcoma	65<br>55	69.2<br>54.5	Zhou et al.,2011<br>Lu et al.,2013
Brain cancer	Glioblastoma	33<br>206<br>77	78.8<br>54.2<br>77.9	Riethdorf et al.,2006<br>Yang et al.,2013<br>Miranda-Gonçalves et al.,2013
Breast cancer	Ductal carcinoma	39<br>1024<br>1285	25.6<br>11.3<br>51.8	Riethdorf et al.,2006<br>Liu et al.,2018<br>Reimers et al.,2004
	Lobular carcinoma	30<br>35<br>245	16.7<br>2.9<br>28.6	Riethdorf et al.,2006<br>Liu et al.,2018<br>Reimers et al.,2004
	Medullary carcinoma	24<br>50	83.3<br>26.0	Riethdorf et al.,2006<br>Liu et al.,2018
Cervical cancer	Adenocarcinoma	6<br>49	50.0<br>69.4	Ju et al.,2008<br>Pinheiro et al.,2009
	Squamous cell carcinoma	23<br>72<br>31	87.0<br>51.4<br>80.6	Riethdorf et al.,2006<br>Ju et al.,2008<br>Pinheiro et al.,2009
Colorectal cancer	Adenocarcinoma	37<br>285<br>328	35.1<br>51.2<br>63.1	Riethdorf et al.,2006<br>Stenzinger et al.,2012<br>Zhu et al.,2013
Esophageal cancer	Squamous cell carcinoma	26<br>217	88.5<br>85.8	Riethdorf et al.,2006<br>Küsters et al.,2022
	Adenocarcinoma	6<br>300	50.0<br>88.3	Riethdorf et al.,2006<br>Küsters et al.,2022
Eye cancer	Retinoblastoma	60	66.7	Adithi et al.,2007
Laryngeal cancer	Squamous cell carcinoma	28<br>48	89.3<br>87.5	Riethdorf et al.,2006<br>Gou et al.,2014
Leukemia	Acute myeloid leukemia	62<br>48	83.9<br>100.0	Fu et al.,2010<br>Spinello et al.,2019
Liver cancer	Hepatocellular carcinoma	24<br>22<br>50	83.3<br>95.4<br>74.0	Riethdorf et al.,2006<br>Mamori et al.,2007<br>Zhu et al.,2015
Lung cancer	Non-small cell carcinoma	127<br>208<br>150<br>208	61.4<br>92.0<br>96.7<br>83.2	Riethdorf et al.,2006<br>Hakuma et al.,2007<br>Sienel et al.,2008<br>Xu et al.,2013
Lymphoma	Hodgkin lymphoma	23<br>34<br>37	43.5<br>100<br>97.3	Riethdorf et al.,2006<br>Thorns et al.,2003<br>Choi et al.,2022
	Non-Hodgkin lymphoma (B cell)	15<br>118	53.3<br>83.0	Riethdorf et al.,2006<br>Choi et al.,2022

	Non-Hodgkin lymphoma (T / NK cell)	36<br>115<br>90	100<br>79.1<br>95.6	Nabeshima et al.,2004<br>Schmidt et al.,2017<br>Choi et al.,2022
Mesothelioma		16<br>9	93.8<br>88.9	Riethdorf et al.,2006<br>Pinheiro et al.,2012
Oral cavity cancer	Squamous cell carcinoma	44<br>74<br>32	95.5<br>75.7<br>100.0	Riethdorf et al.,2006<br>Monteiro et al.,2014<br>Yu et al.,2015
Ovarian cancer	Serous carcinoma	43<br>69<br>177	58.1<br>87.0<br>64.4	Riethdorf et al.,2006<br>Davidson et al.,2003<br>Zhao et al.,2013
	Non-serous carcinoma	50<br>65	64.0<br>64.6	Riethdorf et al.,2006<br>Zhao et al.,2013
Pancreatic cancer	Ductal adenocarcinoma	39<br>108	87.2<br>92.6	Riethdorf et al.,2006<br>Li et al.,2013
Prostate cancer		68<br>120<br>240	54.4<br>65.0<br>47.1	Riethdorf et al.,2006<br>Madigan et al.,2008<br>Zhong et al.,2012
Renal cancer	Clear cell carcinoma	46<br>53<br>322	69.6<br>88.7<br>97.2	Riethdorf et al.,2006<br>Liang et al.,2009<br>Rabien et al.,2013
	Papillary carcinoma	24<br>42	58.3<br>85.7	Riethdorf et al.,2006<br>Rabien et al.,2013
Skin cancer	Melanoma	35<br>150<br>136<br>69	31.4<br>78.0<br>72.1<br>81.2	Riethdorf et al.,2006<br>Chen and Zhu,2010<br>Caudron et al.,2016<br>Liu et al.,2020
Stomach/Gastric Cancer	Diffuse adenocarcinoma	17<br>213<br>140	41.2<br>69.0<br>59.3	Riethdorf et al.,2006<br>Shou et al.,2012<br>Chu et al.,2014
	Intestinal adenocarcinoma	33<br>223<br>83	39.4<br>6.3<br>60.2	Riethdorf et al.,2006<br>Shou et al.,2012<br>Chu et al.,2014
Testicular cancer	Seminoma	44<br>65	72.7<br>81.5	Riethdorf et al.,2006<br>Bi et al.,2012
Thyroid cancer	Follicular carcinoma	43<br>56	16.3<br>66.1	Riethdorf et al.,2006<br>Tan et al.,2008
	Papillary carcinoma	31<br>100	3.2<br>64.0	Riethdorf et al.,2006<br>Tan et al.,2008
Uterine cancer	Endometroid endometrial carcinoma	37<br>107<br>45	40.5<br>44.9<br>64.4	Riethdorf et al.,2006<br>Yuan et al.,2015<br>Latif et al.,2017
	Non-endometroid endometrial carcinoma	14<br>43	64.3<br>64.3	Riethdorf et al.,2006<br>Latif et al.,2017
	Uterine leiomyosarcoma	32	87.5	Kefeli et al.,2016
Vulva cancer	Squamous cell carcinoma	27	92.6	Riethdorf et al.,2006

Supplementary References

Adithi, M., Nalini, V., Kandalam, M., and Krishnakumar, S. (2007). Expression of matrix metalloproteinases and their inhibitors in retinoblastoma. *J Pediatr Hematol Oncol* 29(6)**,** 399-405. doi: 10.1097/MPH.0b013e3180683bf1.

Bi, X.-C., Liu, J.-M., He, H.-C., Ye, Y.-K., Han, Z.-D., Dai, Q.-S., et al. (2012). Extracellular matrix metalloproteinase inducer: a novel poor prognostic marker for human seminomas. *Clin Transl Oncol* 14(3)**,** 190-196. doi: 10.1007/s12094-012-0783-5.

Caudron, A., Battistella, M., Feugeas, J.P., Pages, C., Basset-Seguin, N., Mazouz Dorval, S., et al. (2016). EMMPRIN/CD147 is an independent prognostic biomarker in cutaneous melanoma. *Exp Dermatol* 25(8)**,** 618-622. doi: 10.1111/exd.13022.

Chen, T., and Zhu, J. (2010). Evaluation of EMMPRIN and MMP-2 in the prognosis of primary cutaneous malignant melanoma. *Med Oncol* 27(4)**,** 1185-1191. doi: 10.1007/s12032-009-9357-y.

Choi, J.-W., Lee, Y., Kim, H., Cho, H.Y., Min, S.K., and Kim, Y.-S. (2022). Coexpression of MCT1 and MCT4 in ALK-positive anaplastic large cell lymphoma: diagnostic and therapeutic implications. *Am J Surg Pathol* 46(2)**,** 241-248. doi: 10.1097/pas.0000000000001820.

Choi, J.W., Kim, Y., Lee, J.H., and Kim, Y.S. (2014). Prognostic significance of lactate/proton symporters MCT1, MCT4, and their chaperone CD147 expressions in urothelial carcinoma of the bladder. *Urology* 84(1)**,** 245.e249-215. doi: 10.1016/j.urology.2014.03.031.

Chu, D., Zhu, S., Li, J., Ji, G., Wang, W., Wu, G., et al. (2014). CD147 expression in human gastric cancer is associated with tumor recurrence and prognosis. *PLoS One* 9(6)**,** e101027. doi: 10.1371/journal.pone.0101027.

Davidson, B., Goldberg, I., Berner, A., Kristensen, G.B., and Reich, R. (2003). EMMPRIN (extracellular matrix metalloproteinase inducer) is a novel marker of poor outcome in serous ovarian carcinoma. *Clin Exp Metastasis* 20(2)**,** 161-169. doi: 10.1023/a:1022696012668.

Fu, J., Fu, J., Chen, X., Zhang, Y., Gu, H., and Bai, Y. (2010). CD147 and VEGF co-expression predicts prognosis in patients with acute myeloid leukemia. *Jpn J Clin Oncol* 40(11)**,** 1046-1052. doi: 10.1093/jjco/hyq098.

Gou, X., Chen, H., Jin, F., Wu, W., Li, Y., Long, J., et al. (2014). Expressions of CD147, MMP-2 and MMP-9 in laryngeal carcinoma and its correlation with poor prognosis. *Pathol Oncol Res* 20(2)**,** 475-481. doi: 10.1007/s12253-013-9720-3.

Hakuma, N., Betsuyaku, T., Kinoshita, I., Itoh, T., Kaga, K., Kondo, S., et al. (2007). High incidence of extracellular matrix metalloproteinase inducer expression in non-small cell lung cancers: association with clinicopathological parameters. *Oncology* 72(3-4)**,** 197-204. doi: 10.1159/000112826.

Ju, X.Z., Yang, J.M., Zhou, X.Y., Li, Z.T., and Wu, X.H. (2008). EMMPRIN expression as a prognostic factor in radiotherapy of cervical cancer. *Clin Cancer Res* 14(2)**,** 494-501. doi: 10.1158/1078-0432.Ccr-07-1072.

Kefeli, M., Yildiz, L., Gun, S., Ozen, F.Z., and Karagoz, F. (2016). EMMPRIN (CD147) expression in smooth muscle tumors of the uterus. *Int J Gynecol Cancer* 35(1)**,** 1-7. doi: 10.1097/pgp.0000000000000216.

Küsters, N., Grupp, K., Grass, J.-K., Bachmann, K., Ghadban, T., Uzunoglu, F.G., et al. (2022). CD147 expression lacks prognostic relevance in esophageal cancer. *J Cancer Res Clin Oncol* 148(4)**,** 837-844. doi: 10.1007/s00432-022-03917-2.

Latif, A., Chadwick, A.L., Kitson, S.J., Gregson, H.J., Sivalingam, V.N., Bolton, J., et al. (2017). Monocarboxylate Transporter 1 (MCT1) is an independent prognostic biomarker in endometrial cancer. *BMC Clin Pathol* 17**,** 27. doi: 10.1186/s12907-017-0067-7.

Li, L., Tang, W., Wu, X., Karnak, D., Meng, X., Thompson, R., et al. (2013). HAb18G/CD147 promotes pSTAT3-mediated pancreatic cancer development via CD44s. *Clin Cancer Res* 19(24)**,** 6703-6715. doi: 10.1158/1078-0432.Ccr-13-0621.

Liang, Y.X., He, H.C., Han, Z.D., Bi, X.C., Dai, Q.S., Ye, Y.K., et al. (2009). CD147 and VEGF expression in advanced renal cell carcinoma and their prognostic value. *Cancer Invest* 27(7)**,** 788-793. doi: 10.1080/07357900802709167.

Liu, M., Tsang, J.Y.S., Lee, M., Ni, Y.B., Chan, S.K., Cheung, S.Y., et al. (2018). CD147 expression is associated with poor overall survival in chemotherapy treated triple-negative breast cancer. *J Clin Pathol* 71(11)**,** 1007-1014. doi: 10.1136/jclinpath-2018-205342.

Liu, N., Qi, M., Li, K., Zeng, W., Li, J., Yin, M., et al. (2020). CD147 regulates melanoma metastasis via the NFAT1-MMP-9 pathway. *Pigment Cell Melanoma Res* 33(5)**,** 731-743. doi: https://doi.org/10.1111/pcmr.12886.

Lu, Q., Lv, G., Kim, A., Ha, J.M., and Kim, S. (2013). Expression and clinical significance of extracellular matrix metalloproteinase inducer, EMMPRIN/CD147, in human osteosarcoma. *Oncol Lett* 5(1)**,** 201-207. doi: 10.3892/ol.2012.981.

Madigan, M.C., Kingsley, E.A., Cozzi, P.J., Delprado, W.J., Russell, P.J., and Li, Y. (2008). The role of extracellular matrix metalloproteinase inducer protein in prostate cancer progression. *Cancer Immunol Immunother* 57(9)**,** 1367-1379. doi: 10.1007/s00262-008-0473-x.

Mamori, S., Nagatsuma, K., Matsuura, T., Ohkawa, K., Hano, H., Fukunaga, M., et al. (2007). Useful detection of CD147 (EMMPRIN) for pathological diagnosis of early hepatocellular carcinoma in needle biopsy samples. *World J Gastroenterol* 13(21)**,** 2913-2917. doi: 10.3748/wjg.v13.i21.2913.

Miranda-Gonçalves, V., Honavar, M., Pinheiro, C., Martinho, O., Pires, M.M., Pinheiro, C., et al. (2013). Monocarboxylate transporters (MCTs) in gliomas: expression and exploitation as therapeutic targets. *Neuro Oncol* 15(2)**,** 172-188. doi: 10.1093/neuonc/nos298.

Monteiro, L.S., Delgado, M.L., Ricardo, S., Garcez, F., do Amaral, B., Pacheco, J.J., et al. (2014). EMMPRIN expression in oral squamous cell carcinomas: correlation with tumor proliferation and patient survival. *Biomed Res Int* 2014**,** 905680. doi: 10.1155/2014/905680.

Nabeshima, K., Suzumiya, J., Nagano, M., Ohshima, K., Toole, B.P., Tamura, K., et al. (2004). Emmprin, a cell surface inducer of matrix metalloproteinases (MMPs), is expressed in T-cell lymphomas. *J Pathol* 202(3)**,** 341-351. doi: https://doi.org/10.1002/path.1518.

Pinheiro, C., Longatto-Filho, A., Pereira, S.M., Etlinger, D., Moreira, M.A., Jubé, L.F., et al. (2009). Monocarboxylate transporters 1 and 4 are associated with CD147 in cervical carcinoma. *Dis Markers* 26(3)**,** 97-103. doi: 10.3233/dma-2009-0596.

Pinheiro, C., Longatto-Filho, A., Soares, T.R., Pereira, H., Bedrossian, C., Michael, C., et al. (2012). CD147 immunohistochemistry discriminates between reactive mesothelial cells and malignant mesothelioma. *Diagn Cytopathol* 40(6)**,** 478-483. doi: https://doi.org/10.1002/dc.22821.

Rabien, A., Stephan, C., Kilic, E., Weichert, W., Kristiansen, G., Miller, K., et al. (2013). Renal cell neoplasias: reversion-inducing cysteine-rich protein with Kazal motifs discriminates tumor subtypes, while extracellular matrix metalloproteinase inducer indicates prognosis. *J Transl Med* 11**,** 258. doi: 10.1186/1479-5876-11-258.

Reimers, N., Zafrakas, K., Assmann, V., Egen, C., Riethdorf, L., Riethdorf, S., et al. (2004). Expression of extracellular matrix metalloproteases inducer on micrometastatic and primary mammary carcinoma cells. *Clin Cancer Res* 10(10), 3422-3428. doi: 10.1158/1078-0432.Ccr-03-0610.

Riethdorf, S., Reimers, N., Assmann, V., Kornfeld, J.W., Terracciano, L., Sauter, G., et al. (2006). High incidence of EMMPRIN expression in human tumors. *Int J Cancer* 119(8), 1800-1810. doi: 10.1002/ijc.22062.

Schmidt, J., Bonzheim, I., Steinhilber, J., Montes-Mojarro, I.A., Ortiz-Hidalgo, C., Klapper, W., et al. (2017). EMMPRIN (CD147) is induced by C/EBPβ and is differentially expressed in ALK+ and ALK− anaplastic large-cell lymphoma. *Lab Invest* 97(9), 1095-1102. doi: 10.1038/labinvest.2017.54.

Shou, Z.X., Jin, X., and Zhao, Z.S. (2012). Upregulated expression of ADAM17 is a prognostic marker for patients with gastric cancer. *Ann Surg* 256(6), 1014-1022. doi: 10.1097/SLA.0b013e3182592f56.

Sienel, W., Polzer, B., Elshawi, K., Lindner, M., Morresi-Hauf, A., Vay, C., et al. (2008). Cellular localization of EMMPRIN predicts prognosis of patients with operable lung adenocarcinoma independent from MMP-2 and MMP-9. *Mod Pathol* 21(9), 1130-1138. doi: 10.1038/modpathol.2008.102.

Spinello, I., Saulle, E., Quaranta, M.T., Pasquini, L., Pelosi, E., Castelli, G., et al. (2019). The small-molecule compound AC-73 targeting CD147 inhibits leukemic cell proliferation, induces autophagy and increases the chemotherapeutic sensitivity of acute myeloid leukemia cells. *Haematologica* 104(5), 973-985. doi: 10.3324/haematol.2018.199661.

Stenzinger, A., Wittschieber, D., von Winterfeld, M., Goeppert, B., Kamphues, C., Weichert, W., et al. (2012). High extracellular matrix metalloproteinase inducer/CD147 expression is strongly and independently associated with poor prognosis in colorectal cancer. *Hum Pathol* 43(9), 1471-1481. doi: 10.1016/j.humpath.2011.10.023.

Tan, H., Ye, K., Wang, Z., and Tang, H. (2008). CD147 expression as a significant prognostic factor in differentiated thyroid carcinoma. *Transl Res* 152(3), 143-149. doi: 10.1016/j.trsl.2008.07.005.

Thorns, C., Bernd, H.W., Hatton, D., Merz, H., Feller, A.C., and Lange, K. (2003). Matrix-metalloproteinases in Hodgkin lymphoma. *Anticancer Res* 23(2b), 1555-1558.

Xu, X.Y., Lin, N., Li, Y.M., Zhi, C., and Shen, H. (2013). Expression of HAb18G/CD147 and its localization correlate with the progression and poor prognosis of non-small cell lung cancer. *Pathol Res Pract* 209(6), 345-352. doi: 10.1016/j.prp.2013.02.015.

Xue, Y.J., Lu, Q., and Sun, Z.X. (2011). CD147 overexpression is a prognostic factor and a potential therapeutic target in bladder cancer. *Med Oncol* 28(4), 1363-1372. doi: 10.1007/s12032-010-9582-4.

Yang, M., Yuan, Y., Zhang, H., Yan, M., Wang, S., Feng, F., et al. (2013). Prognostic significance of CD147 in patients with glioblastoma. *J Neuro Oncol* 115(1), 19-26. doi: 10.1007/s11060-013-1207-2.

Yu, Y.H., Morales, J., Feng, L., Lee, J.J., El-Naggar, A.K., and Vigneswaran, N. (2015). CD147 and Ki-67 overexpression confers poor prognosis in squamous cell carcinoma of oral tongue: a tissue microarray study. *Oral Surg Oral Med Oral Pathol Oral Radiol* 119(5), 553-565. doi: 10.1016/j.oooo.2014.12.022.

Yuan, Y., Shen, N., Yang, S.Y., Zhao, L., and Guan, Y.M. (2015). Extracellular matrix metalloproteinase inducer and matrix metalloproteinase-2 overexpression is associated with loss of hormone receptor expression and poor prognosis in endometrial cancer. *Oncol Lett* 10(1), 342-348. doi: 10.3892/ol.2015.3177.

Zhao, Y., Chen, S., Gou, W.F., Niu, Z.F., Zhao, S., Xiao, L.J., et al. (2013). The role of EMMPRIN expression in ovarian epithelial carcinomas. *Cell Cycle* 12(17), 2899-2913. doi: 10.4161/cc.25950.

Zhong, W.D., Liang, Y.X., Lin, S.X., Li, L., He, H.C., Bi, X.C., et al. (2012). Expression of CD147 is associated with prostate cancer progression. *Int J Cancer* 130(2), 300-308. doi: 10.1002/ijc.25982.

Zhou, Q., Zhu, Y., Deng, Z., Long, H., Zhang, S., and Chen, X. (2011). VEGF and EMMPRIN expression correlates with survival of patients with osteosarcoma. *Surg Oncol* 20(1), 13-19. doi: 10.1016/j.suronc.2009.09.002.

Zhu, S., Chu, D., Zhang, Y., Wang, X., Gong, L., Han, X., et al. (2013). EMMPRIN/CD147 expression is associated with disease-free survival of patients with colorectal cancer. *Med Oncol* 30(1), 369. doi: 10.1007/s12032-012-0369-7.

Zhu, S., Li, Y., Zhang, Y., Wang, X., Gong, L., Han, X., et al. (2015). Expression and clinical implications of HAb18G/CD147 in hepatocellular carcinoma. *Hepatol Res* 45(1), 97-106. doi: 10.1111/hepr.12320.
